# Enhancing Self-Healing Performance of Cement-Based Materials Through Sodium Silicate and SAP Composite Incorporation

**DOI:** 10.3390/ma19061249

**Published:** 2026-03-21

**Authors:** Yumei Kang, Rongbao Wu, Yu Qiao, Chang Xu

**Affiliations:** 1 School of Resources and Civil Engineering, Northeastern University, 3-11 Wenhua Road, Heping District, Shenyang 110819, China; 2 Jintai Chemical Industrial Co., Ltd., Tongling 244100, China

**Keywords:** cement mortar, self-healing performance, compressive strength recovery rate, capillary water absorption, ultrasonic pulse velocity

## Abstract

Conventional admixture-based self-healing technologies are often limited by inadequate internal water supply and a scarcity of unhydrated gel particles. Therefore, this study proposes a new self-healing method that leverages the synergistic interplay between the chemical repair of sodium silicate and the physical clogging of superabsorbent polymers (SAPs) to overcome the aforementioned limitations. The healing efficiency of cement mortar was assessed through compressive strength recovery, capillary water absorption, and ultrasonic pulse velocity (UPV). Microstructural evolution and healing mechanisms were elucidated using scanning electron microscopy (SEM) and X-ray diffraction (XRD). Results indicate that at an optimal dosage (0.5 wt.% for both admixtures), the healing performance is significantly enhanced: the compressive strength recovery rate reaches 103.1%, the capillary water absorption coefficient decreases by 16.57 × 10^−3^, and the UPV recovery achieves 95.4%. Microstructural analysis reveals that sodium silicate facilitates the reaction between ${\mathrm{Ca}}^{2+}$ and ${SiO}_{3}^{2-}$ ions, leading to the in situ precipitation of dense C-S-H gel at the crack interface, thereby enabling chemical repair. In contrast, SAP contributes to physical sealing via a swelling and release mechanism.

## 1. Introduction

Cementitious materials are the core structural components in modern construction. The initiation and propagation of cracks within these materials are primary contributors to the degradation of structural durability and pose significant operational safety risks. Corrosive agents infiltrate through these cracks, inducing secondary deterioration processes such as reinforcement corrosion and freeze–thaw damage. This phenomenon constitutes a major constraint on the service life of concrete structures [1,2,3,4,5]. Consequently, some researchers have turned to addressing the issue from the material’s composition, incorporating repair agents into the cement matrix to promote crack healing and thereby improve cracking resistance [6,7,8]. Effective crack management in concrete structures relies on routine inspection, maintenance, and repair. However, the efficacy of these measures is severely hampered when cracks are concealed or difficult to access [9]. Moreover, structural repairs incur significant costs and impose broader societal burdens, amplifying the resultant economic strain [10].

Self-healing technology offers a promising solution to crack management in cementitious materials, enabling cost reduction and extended service life without manual repairs [11,12]. However, autogenous self-healing, primarily through continued hydration and calcium carbonate precipitation, is inherently limited to cracks below approximately 200 μm [13,14]. To overcome this limitation, various engineered self-healing strategies have been developed, including microencapsulation of healing agents [15,16,17], microbial-induced calcite precipitation [18,19], and the use of admixtures [20,21]. For instance, Ma et al. [22] reported that microbial self-healing concrete using expanded perlite as a carrier achieved a 29.51% strength recovery after 28 days, while Sun et al. [23] demonstrated that polydopamine-coated isocyanate microcapsules could effectively heal cracks up to 100 μm in cement paste. Despite their effectiveness, both approaches face practical limitations: microorganisms are constrained by cost and carrier suitability [23], and microcapsules involve complex manufacturing and high costs [24]. Therefore, there is an urgent need to develop a new type of self-healing material that strikes a balance between cost and healing performance.

In this context, SAP and sodium silicate have attracted considerable interest owing to their distinct advantages in internal curing and chemical reactivity. SAP can absorb and retain large amounts of water, subsequently releasing it during drying periods, thereby promoting continued hydration and providing physical crack sealing upon swelling [25,26]. Lyu et al. [27] explored the impact of SAP particle size and content, demonstrating their critical role in regulating healing efficiency. Concurrently, Formagini et al. [28] provided a comprehensive evaluation of SAP’s mechanical self-healing contribution in both conventional and high-performance concrete, confirming its efficacy across different matrix types. These studies collectively indicate that while SAP is effective, its performance is highly dependent on its intrinsic properties and the exposure environment [29]. In parallel, sodium silicate has been extensively studied as a chemical healing agent. Sodium silicate can react with calcium ions released from cement hydration to form C-S-H gel which can chemically seal cracks and improve matrix density [30]. For example, Mascarenhas et al. [31] reported that sodium silicate-encapsulated microcapsules successfully healed cracks in cement paste. Ozen and Stephan [32] broadened the application scope by impregnating perlite with sodium silicate for use in geopolymers, showing its potential beyond traditional Portland cement systems.

The two approaches may offer significant complementary potential in terms of physical sealing and chemical repair of cracks. However, existing studies have predominantly investigated these materials individually [27,33], with a lack of systematic understanding regarding their synergistic effects. This research gap is particularly pronounced when it comes to applications involving different maintenance environments.

This study combines theoretical analysis with experimental investigation to systematically evaluate the self-healing capacity of cementitious matrices incorporating sodium silicate and SAP under both dry and wet curing regimes. Through tests of compressive strength recovery, UPV, and capillary water absorption, the influence of varying admixture dosages on the macro-scale healing performance was quantitatively assessed. Furthermore, SEM and XRD were used to examine microstructural evolution in healed crack regions and to identify the composition of healing products, thereby elucidating the underlying mechanisms.

## 2. Materials and Methods

### 2.1. Raw Materials

**Figure 1:**
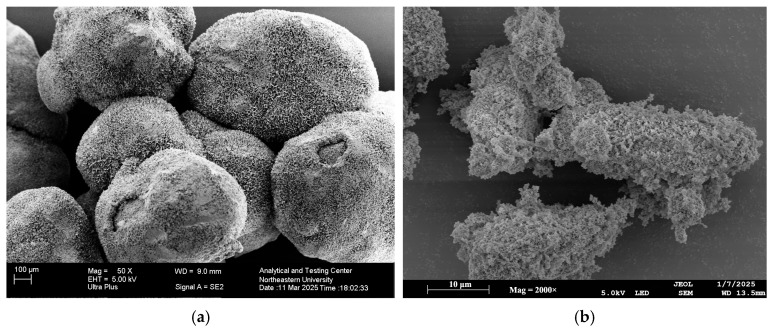
Microstructure diagram of admixtures: (**a**) Sodium silicate; (**b**) SAP.

The raw materials used in this study included: P.O 42.5 ordinary Portland cement (Shenyang, China); SAP (Shanghai Funa New Materials Technology Co., Ltd., Shanghai, China) with a particle size of 100–120 mesh (properties listed in Table 1); analytical grade (AR) sodium silicate (Na_2_SiO_3_·5H_2_O), manufactured by Zhiyuan Chemical Reagents Co., Ltd. in Tianjin, China; natural river sand (Shenyang, China) with a fineness modulus of 2.78 graded as Zone II medium sand; and a high-efficiency water-reducing agent (Shandong Wanshan Chemical Co., Ltd., Weifang, China), achieving a water reduction rate of 20% at a dosage of 2%. The microstructures of the sodium silicate and SAP were presented in Figure 1.

### 2.2. Mix Proportions

A total of twelve mix designs were formulated. The SAP content was set at 0, 0.5, and 1.0% by mass of cement, designated as A0, A5, and A10, respectively. The sodium silicate dosage was set at 0, 0.5, 1.0, and 1.5% by mass of cement, labeled as S0, S5, S10, and S15, respectively. Each compound mixture group was named accordingly; for example, S5A5 denoted sodium silicate and SAP dosages both at 0.5%. A plain cement mortar (CG) served as the control. The detailed mix proportions were summarized in Table 2. To maintain consistent workability across all mixtures, the mixing water content was slightly adjusted as needed, compensating for the water absorption by SAP and the rheological influence of sodium silicate.

### 2.3. Sample Preparation and Test Methods

#### 2.3.1. Sample Preparation and Pre-Damage

**Figure 2:**
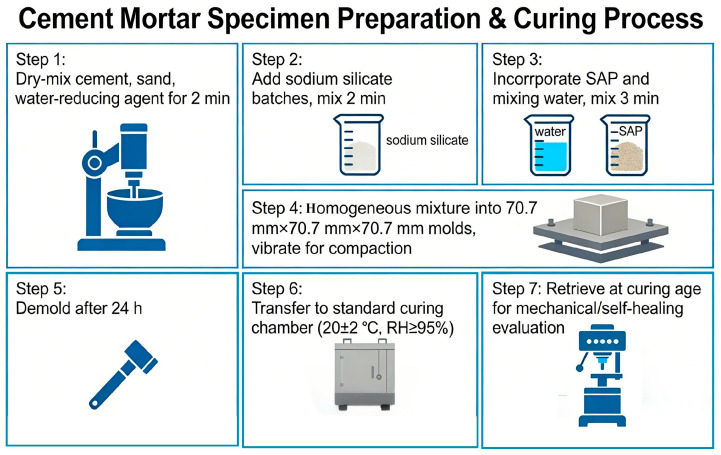
Flowchart for preparing experimental specimens.

The dimensions of all cement mortar specimens used in the experiment were 70.7 mm × 70.7 mm × 70.7 mm. During specimen preparation, cement, sand, and the water-reducing agent were first dry-mixed for 2 min. Sodium silicate was then added in batches and mixed for an additional 2 min. Finally, SAP and mixing water were incorporated, followed by 3 min of further mixing. The homogeneous mixture was cast into molds and vibrated for compaction. After 24 h, the specimens were demolded and transferred to a standard curing chamber (20 ± 2 °C, RH ≥ 95%). Upon reaching the designated curing age, the specimens were retrieved for mechanical and self-healing evaluation. The experimental specimen preparation flowchart is shown in Figure 2.

Pre-damage was induced via uniaxial compression at a loading rate of 0.5 kN/s. After 28 d of standard curing, specimens intended for self-healing assessment were pre-loaded to 75% of their ultimate compressive strength, at which point loading was ceased. This damage level was selected to simulate severe service damage in real-world concrete structures, where microcracks have propagated and coalesced into dominant macrocracks—resembling the state of structures after extreme events or long-term deterioration [26,34,35]—without reaching ultimate failure.

#### 2.3.2. Self-Healing Maintenance Environment

Two curing regimes were adopted to simulate distinct service environments: a dry condition (RH ≤ 45%, 28 ± 2 °C) representing hot and arid climates, and a damp condition (RH ≥ 90%, 28 ± 2 °C) simulating a high-humidity air environment. Pre-cracked specimens were placed in the respective environments and subjected to healing periods of 14 and 28 days.

#### 2.3.3. Compressive Strength Testing

Compressive strength testing shall be conducted in accordance with the Standard Test Methods for Basic Properties of Building Mortars JGJ/T70-2009 [36] (similar test methods refer to ASTM C109/C109M [37]). Specimen dimensions shall be uniformly 70.7 mm × 70.7 mm × 70.7 mm. After standard curing for 28 days at a temperature of 20 ± 2 °C and relative humidity ≥ 95%, compressive strength testing shall be performed. At least three samples shall be tested under each set of working conditions, ensuring that each data point represents the average of three independent samples, with error bars corresponding to the standard deviation.

#### 2.3.4. Self-Healing Compressive Strength Testing

Following the dry and wet curing periods (14 and 28 days), both the pre-damaged and intact specimens from each group were tested again for compressive strength. The compressive strength recovery rate, $\eta_{1}$, was calculated for each group using Equation (1):

(1) $$\eta_{1}\;={\;F}_{rec}\;/{\;F}_{0}\;\times \;100\%\;$$

where ${\;F}_{rec}$ is the compressive strength (MPa) of the pre-damaged specimen after healing, and ${\;F}_{0}$ is the compressive strength (MPa) of the intact specimen at the same age.

#### 2.3.5. Capillary Absorption Testing

Capillary water absorption was tested according to ASTM C1585-13 [38]. Specimens were first dried to constant mass at 60 °C. Their sides were then sealed with epoxy resin, leaving only the top and bottom surfaces exposed to water. The specimens were placed on supports and immersed in water to a depth of 3–5 mm. The capillary absorption rate *I* was calculated using Equation (2):

(2) $$I\;=\;∆m_{t}\;/\;A\rho \;$$

where $∆m_{t}$ is the change in mass (kg) of the specimen after immersion time t, A is the exposed surface area (m^2^); and ρ the density of water (1000 kg/m^3^). Mass readings were taken at t = 0, 5, 15, 30, 60, 120,180 min using a balance with a precision of 0.01 g.

#### 2.3.6. UPV Recovery Rate Testing

UPV was measured using a non-metallic ultrasonic tester (ZBL-U5200, Beijing Zhibolian Technology Co., Ltd., Beijing, China) equipped with 50 kHz transverse wave transducers. The wavelength range of the ultrasonic pulses employed is approximately 60 mm to 90 mm. To ensure consistent acoustic coupling, a thin layer of petroleum jelly was applied between the transducer faces and the specimen surfaces. The UPV recovery rate λ was calculated to evaluate the healing extent according to Equation (3):

(3) $$\lambda \;=U_{rce}\;/\;U_{0}\times 100\%$$

where $U_{rce}$ is the UPV (m/s) of the healed specimen after pre-damage, and $U_{0}$ is the UPV (m/s) of the intact reference specimen at the same age.

#### 2.3.7. Microanalysis

SEM and XRD were used to analyze the microstructure and phase composition of the specimens. These techniques characterized the pore structure and the distribution of healing products, elucidating how sodium silicate and SAP influence the mechanical and self-healing behavior of the mortar.

For SEM, samples were extracted from crack-healed zones showing evident deposition of healing products. Specimens were immersed in anhydrous ethanol for 24 h to terminate hydration, then dried in a 60 °C oven to constant weight. Prior to examination, the specimen surfaces were coated with gold leaf.

For XRD, samples were similarly collected from healed regions, ground into fine powder, and stored in sealed glass vials. For measurement, the powder was placed on a clean glass slide and analyzed using an X-ray diffractometer over a 2θ range of 5° to 70° with a scanning step of 0.02° and a scanning speed of 6.5°/min for phase identification.

## 3. Results and Discussion

### 3.1. Compressive Strength

**Figure 3:**
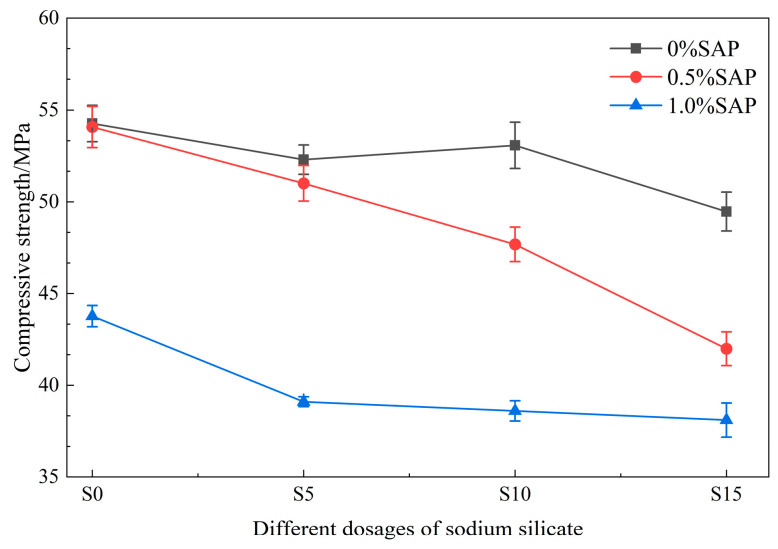
Changes in compressive strength of specimens with the dosage of repair agent.

After 28 days of standard curing, the compressive strength of each group is presented in Figure 3. Specimens containing only sodium silicate exhibited lower strength than the control group (CG), with decreases of 3.63%, 2.19%, and 8.84% corresponding to sodium silicate dosages of 0.5%, 1.0%, and 1.5%, respectively. For SAP-only mixtures, the strength decreased marginally by 0.36% at 0.5% SAP, but dropped notably by 19.35% at 1.0% SAP. In composites incorporating both materials, compressive strength decreased as the dosage of either admixture increased. All mixes containing 1.0% SAP exhibited strengths below 42.5 MPa, which may fall short of some structural requirements. Overall, higher contents of sodium silicate and SAP led to reduced compressive strength, indicating that dosage must be carefully controlled in practical applications.

Therefore, controlling the dosage of both admixtures is crucial to mitigate strength reduction [29]. At optimal levels, sodium silicate and SAP act synergistically: SAP provides internal curing to sustain cement hydration, while sodium silicate catalyzes the formation of C-S-H and other hydration products. These products fill micro-voids in the matrix and occupy spaces created by SAP after water release, collectively contributing, along with portlandite Ca(OH)_2_, to a denser microstructure [32,33,36]. However, excessive dosages of either material can disrupt normal hydration, impair microstructural integrity, and consequently reduce macroscopic mechanical performance [38,39]. Hence, to preserve mortar strength, the content of both sodium silicate and SAP must be strictly controlled.

### 3.2. Compressive Strength Recovery Rate

**Figure 4:**
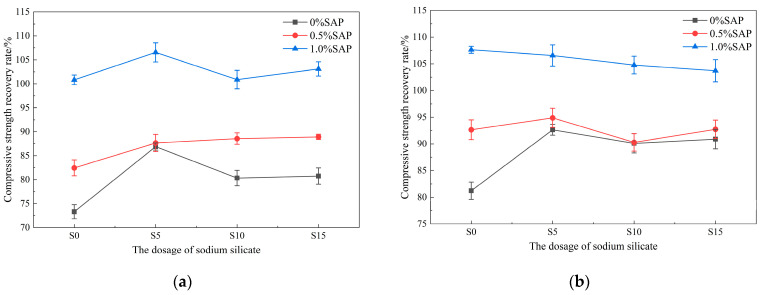
Compressive strength recovery rate of specimens cured for 14 days under different curing conditions: (**a**) Dry; (**b**) Damp.

**Figure 5:**
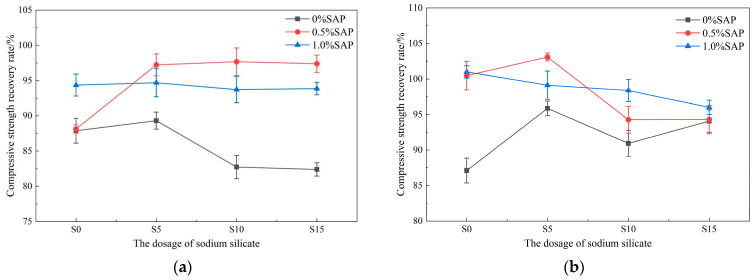
Compressive strength recovery rate of specimens cured for 28 days under different curing conditions: (**a**) Dry; (**b**) Damp.

The compressive strength recovery rates of pre-damaged mortar after 14 and 28 days of healing under different curing conditions are presented in Figure 4 and Figure 5. Specimens without SAP showed the lowest recovery. At 14 days, mixes with 1.0% SAP consistently achieved higher recovery than those with 0.5% SAP. This is attributed to the additional water released by SAP during early hydration, which accelerates hydration reactions and facilitates crack closure [40]. However, at 28 days, under uniform sodium silicate dosage, recovery in some of the 0.5% SAP mixes surpassed that of the 1.0% group, suggesting that excessive SAP may adversely affect the strength of the matrix in later stages. In contrast, an appropriate SAP content, in sustained synergy with sodium silicate, provides better long-term healing. Overall, the combination of 0.5% SAP and 0.5% sodium silicate yielded the strongest self-healing performance.

The healing environment plays a critical role in the self-healing mechanism. Under dry conditions and with 0.5% SAP, the compressive strength recovery rate increased with sodium silicate content, although the rate of increase diminished when the content rose from 1.0% to 1.5%. In high-humidity environments, after 28 days of curing, however, specimens containing 0.5% SAP showed a recovery rate that first increased and then decreased with increasing sodium silicate, peaking at 103.1 ± 0.56% for the 0.5% sodium silicate dosage. This suggests that excessively high sodium silicate may disturb the pH balance of the system, hindering the deposition of gel. Recovery rates in high-humidity conditions were consistently higher than in dry settings, reaffirming that moisture availability is a central limiting factor for autogenous healing [25]. In dry environments, sodium silicate alone provided only limited improvement in strength recovery, and excessive dosages even reduced it. In contrast, the combined use of SAP markedly enhanced recovery, demonstrating that SAP effectively mitigates moisture deficiency in arid conditions through internal humidity regulation [39]. Thus, the moisture-supplying function of SAP is essential to the performance of the dual-admixture system.

### 3.3. Capillary Water Absorption Rate

**Figure 6:**
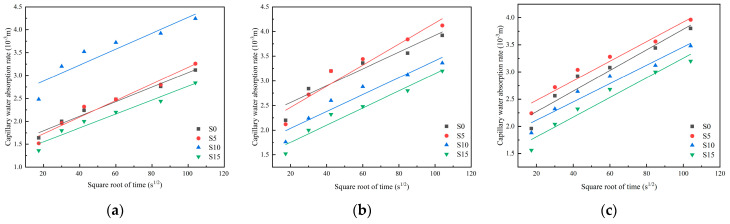
Capillary water absorption of cement mortar specimens after 14-Day self-healing: (**a**) 0% SAP; (**b**) 0.5% SAP; (**c**) 1.0% SAP.

**Figure 7:**
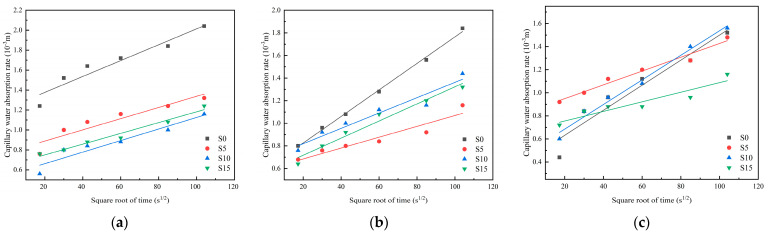
Capillary water absorption of cement mortar specimens after 28-Day self-healing: (**a**) 0% SAP; (**b**) 0.5% SAP; (**c**) 1.0% SAP.

Capillary water absorption of pre-damaged mortar at different curing ages is shown in Figure 6 and Figure 7. The effect of sodium silicate and SAP on the capillary water absorption coefficient (the slope of the straight lines, Figure 6 and Figure 7) of cement mortar shows significant time dependence. After 14 days of curing, all mixtures showed a capillary water absorption coefficient statistically similar to the control. By 28 days, however, the blend containing 0.5% sodium silicate and 0.5% SAP (S5A5) demonstrated a markedly lower capillary water absorption coefficient, reduced by 16.57 × 10^−3^ compared to the control group, indicating superior impermeability. This delayed response suggests that the healing agents require time to become effective after crack formation. With prolonged curing, their repair action progresses—sealing cracks and obstructing water-transport pathways—thereby enhancing the overall impermeability of the material.

The effect of dosage on the capillary water absorption coefficient differed between sodium silicate and SAP, and this difference grew more distinct with longer curing. At 14 days’ curing, the capillary water absorption coefficient of test groups with sodium silicate dosages of 0.5%, 1.0% and 1.5% were all close to the control group; however, at 28 days’ curing, they were significantly lower than the control group. This pattern can be explained by the fact that although reduced cracking diminishes pathways for capillary water absorption coefficient, both the chemical reaction of sodium silicate and the physical action of SAP moisture-driven processes [41,42]. Initially, the presence of these agents may even slightly elevate the matrix’s water adsorption capacity, leading to a comparable capillary water absorption coefficient at an early age. By 28 days of curing, sodium silicate and SAP had repaired most cracks, hindering water molecule penetration into the cement matrix and significantly reducing the capillary water absorption coefficient. Notably, even specimens with sodium silicate alone displayed a lower capillary water absorption coefficient than the control at 28 days, confirming its contribution to self-healing.

### 3.4. UPV Recovery Rate

**Figure 8:**
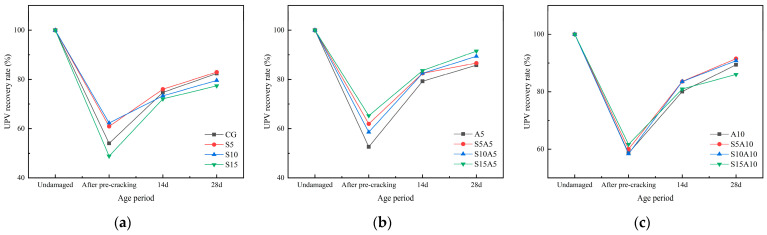
UPV recovery rate as a function of healing agent dosage in mortar specimens under dry conditions: (**a**) 0% SAP; (**b**) 0.5% SAP; (**c**) 1.0% SAP.

**Figure 9:**
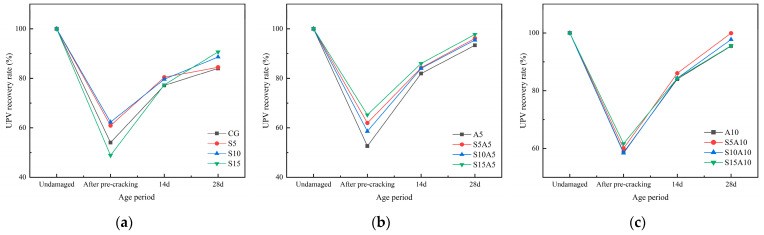
UPV recovery rate as a function of healing agent dosage in mortar specimens under high-humidity conditions: (**a**) 0% SAP; (**b**) 0.5% SAP; (**c**) 1.0% SAP.

The UPV recovery rates of cement mortar specimens under different repair agent dosages and curing environments are shown in Figure 8 and Figure 9. Both the dosage of healing agents and the curing environment significantly influenced the healing outcome. All specimens incorporating both repair agents achieved higher UPV recovery than the plain control (CG). Under high-humidity curing conditions, the UPV recovery rates of mortar specimens after 28 days of self-healing were generally higher than those in dry environments. After pre-loading, UPV values dropped by approximately 40%, followed by a rapid initial recovery that gradually slowed; recovery reached about 90% after 14 days and approached 100% after 28 days. Regarding dosage effects, the 1.0% SAP group showed no clear advantage over the 0.5% SAP group in UPV recovery. For sodium silicate, the 0.5% dosage consistently yielded higher recovery than the control at both ages, whereas recovery in other dosage groups varied with SAP content.

The ultrasonic pulse velocity (UPV) is positively correlated with the internal healing extent of the matrix [43]. As healing proceeds, cement hydration progressively advances, enabling the material’s self-healing properties to take effect. Internal voids within the matrix are gradually filled by hydration products or water-absorbed, expanded SAP, leading to a denser structure. This facilitates a transition from diffraction to transmission during ultrasonic propagation, effectively shortening the propagation path and thereby increasing the wave velocity [44]. However, the self-healing capacity is finite. As unhydrated gel particles are gradually depleted and the pore structure of the matrix progressively densifies, the reaction driving force within the system weakens [45]. Consequently, the generation rate of self-healing products decreases, leading to a diminished increase in the UPV recovery rate.

### 3.5. Microstructural Analysis

#### 3.5.1. X-Ray Diffraction Analysis

**Figure 10:**
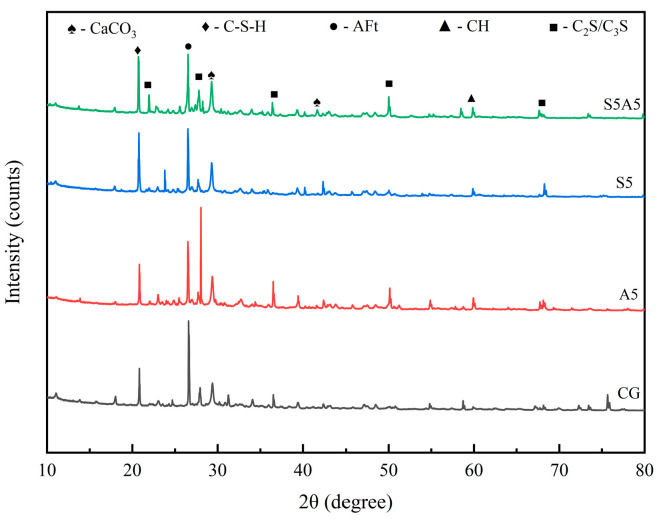
XRD images of hydration products of each group of mortar specimens during 28 days of repair.

XRD was performed to examine the phase composition of cement mortar after 28 days of healing under high-humidity conditions, comparing control (CG), sodium silicate-only (S5), SAP-only (A5), and dual-admixture (S5A5) specimens. The XRD patterns are presented in Figure 10. The diffraction peaks in the patterns correspond to the primary hydration products in the samples, including unhydrated cement clinker (C2S/C3S), hydrated calcium sulfoaluminate (AFt), and calcium hydroxide crystals (CH). Distinct C2S/C3S peaks were observed in all specimens, attributed to the appropriate water-cement ratio providing sufficient moisture for hydration reactions [46,47]. As observed in Figure 10, the peak intensity for unhydrated cement clinker was highest in the SAP-only sample, indicating that SAP exerts a certain inhibitory effect on cement hydration during water absorption [48]. This intensity decreased with sodium silicate addition, indicating that sodium silicate promotes hydration. These findings align with the trends observed in macroscopic performance.

Analysis of the composite specimens revealed enhanced diffraction peaks for CaCO_3_. This is because, during the healing process, sodium silicate promotes the dissolution of calcium ions and facilitates carbonate precipitation within the crack zone [32]. Compared to specimens in the single-SAP admixture group, the peak intensities of C2S or C3S in the dual-admixture group specimens were reduced, indicating that C2S or C3S crystals within the matrix were disrupted during the self-healing process. Concurrently, the C-S-H peak showed increased intensity, confirming that sodium silicate promotes the dissolution of C2S/C3S and enhances the formation of C-S-H gel. The presence of additional diffraction peaks in the dual-blended specimen (S5A5) indicates more complete hydration and a more complex product composition. Furthermore, a new diffraction peak corresponding to N-S-H (NaHSiO_3_·3H_2_O) was detected in specimens containing sodium silicate (Figure 10). This hygroscopic and expansive gel can, at high dosages, induce swelling in humid environments, thereby disrupting the microstructural integrity and compromising the self-healing performance [49].

#### 3.5.2. SEM Analysis

**Figure 11:**
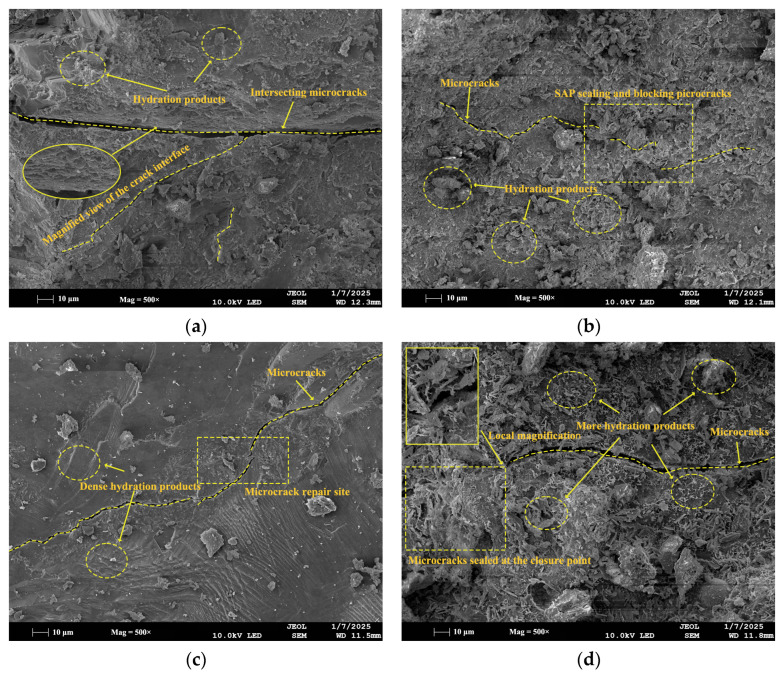
SEM characterization of self-healing products in cement mortar: (**a**) CG; (**b**) A5; (**c**) S5; (**d**) A5S5.

SEM was performed to examine the microstructure of mortar specimens from the control (CG), single-admixture (S5, A5), and dual-admixture (S5A5) groups after 28 days of healing. As shown in Figure 11a, the control specimen displayed elongated cracks and a porous structure, characteristic of shrinkage-induced cracking [50,51]. In the SAP-only specimen (Figure 11b), swollen SAP particles released moisture into microcracks, promoting further hydration of unreacted cement and the formation of plate-like or fibrous products that partially sealed the interface. However, shrunken SAP residues left behind voids, resulting in localized structural loosening. Specimens with only sodium silicate (Figure 11c) showed a relatively uniform and dense C-S-H gel morphology. In the dual-admixture system (S5A5, Figure 11d), some SAP particles remained swollen, while the surrounding matrix developed a more abundant and inter-grown network of hydration products, predominantly in lamellar and fibrous forms. This microstructure demonstrates that the combination of sodium silicate and SAP synergistically enhances crack-interface restoration.

Microscopic examination of the S5A5 specimen (Figure 11d) revealed a marked increase in healing products, a reduction in fissures, and a denser surface compared to other groups. The hydration products exhibited diverse morphologies, including lamellar Ca(OH)_2_ crystals and intergrown C-S-H gel. Spherical-like particles were observed encapsulated and aggregated by hydration products in the vicinity of microcracks. Owing to local variations in reactant concentration and distribution, these encapsulated particles displayed irregular shapes. Regions enriched with calcium ions on particle surfaces acted as nucleation sites, promoting the progressive accumulation of hydration products [52]. As healing advanced, these products bonded with the surrounding matrix, forming integrated composite microstructures. These unevenly distributed and morphologically diverse hydration products bound the particles into cohesive aggregates, some of which even seal microcracks to inhibit further propagation. Consequently, the blended system exhibits superior macroscopic properties during self-healing, including higher compressive strength recovery, lower capillary water absorption, and enhanced UPV recovery.

In summary, the internal curing provided by SAP ensures a sustained moisture supply for the sodium silicate reaction, creating favorable conditions for C-S-H gel formation—especially in dry environments. Concurrently, the hydration products resulting from the sodium silicate reaction encapsulate and immobilize SAP particles, while also filling the voids left after SAP water release, thereby reducing the structural defects associated with SAP incorporation. This synergy between moisture regulation and chemical activation is key to the enhanced self-healing performance of the combined admixture system.

## 4. Conclusions

(1) The combined use of sodium silicate and SAP reduces the compressive strength of cement mortar, but this adverse effect can be minimised when the addition rate is ≤0.5%.
(2) Owing to the synergistic effect of sodium silicate and SAP, the optimal combination (0.5% each) achieved a compressive strength recovery of 103.1%, a UPV recovery rate of 95.4%, and a reduction in capillary water absorption coefficient of 16.57 × 10^−3^, demonstrating significantly enhanced self-healing performance.
(3) Under RH ≥ 90% conditions, compressive strength recovery rates reached 103.1% after 28 days. In dry environments, SAP-containing specimens achieved recovery rates up to 91.3%, comparable to those of non-SAP specimens under high humidity.
(4) Microstructural analysis indicates that the composite admixture promotes more complete hydration and yields a broader range of healing products. These products densify the interfacial zone and enhance crack-sealing capability.

This study confirms the potential of the sodium silicate–SAP system to improve the self-healing properties of cementitious materials. Subsequent work should focus on long-term durability and field performance under adverse conditions, including material behaviour under dynamic and repeated loading. Concurrently, bender elements may be employed in the future to measure shear wave velocities, thereby ensuring improved self-healing performance evaluation. Cyclic triaxial testing is recommended to evaluate the self-healing efficiency and mechanical properties of this composite for geotechnical applications.

## Figures and Tables

**Table 1:** SAP performance metrics.

pH	Moisture Content (%)	Mass Flow (g/s)	Apparent Density (g/cm^3^)	Water Absorption Rate (s)
5.9	3.7	11.3	0.7	23

**Table 2:** Specimen mix ratio.

Group	Sodium Silicate Dosage (%)	SAP (%)	Cement (kg/m^3^)	Water (kg/m^3^)	Sand (kg/m^3^)	Water Reducer (kg/m^3^)
CG	0	0	450	180	1300	9
A5	0	0.5	448	180	1300	9
A10	0	1	445	180	1300	9
S5	0.5	0	448	180	1300	9
S5A5	0.5	0.5	445	180	1300	9
S5A10	0.5	1	443	180	1300	9
S10	1	0	445	180	1300	9
S10A5	1	0.5	443	180	1300	9
S10A10	1	1	440	180	1300	9
S15	1.5	0	443	180	1300	9
S15A5	1.5	0.5	440	180	1300	9
S15A10	1.5	1	438	180	1300	9

## Data Availability

The original contributions presented in this study are included in the article. Further inquiries can be directed to the corresponding author.

## Abbreviations

The following abbreviations are used in this manuscript:

SAP	Superabsorbent polymer
UPV	Ultrasonic pulse velocity
SEM	Scanning electron microscopy
XRD	X-ray diffraction
